# Fatty acids characterization and oxidative stability of spray dried designer egg powder

**DOI:** 10.1186/s12944-018-0931-1

**Published:** 2018-12-13

**Authors:** Amna Javed, Muhammad Imran, Nazir Ahmad, Abdullah Ijaz Hussain

**Affiliations:** 1 Department of Food Science, Nutrition and Home Economics, Government College University, Faisalabad, Pakistan; 2 Institute of Home and Food Sciences, Faculty of Life Sciences, Government College University, Faisalabad, Pakistan; 3 Department of Chemistry, Faculty of Physical Sciences, Government College University, Faisalabad, Pakistan

**Keywords:** Lipid oxidation, Storage stability, Fatty acids profile, Omega eggs, Spray drying

## Abstract

**Background:**

Designer eggs (DEs) have gained positive importance in maintaining cholesterol level, triglyceride profile and protection towards cardiovascular diseases due to the presence of essential fatty acids (EFAs) such as omega-3 (or) n-3 fatty acids. However, extreme heat conditions effect the quality as well as quantity of EFAs during the production of designer egg dried powder (DEDP). Therefore, the main mandate of research was the development of DEDP and determination of spray drying conditions impact on fatty acids composition of DEDP samples.

**Methods:**

The DEs were produced, collected, de-shelled, homogenized and diluted before spray drying to get fine powder. The spray drying of DEs was carried out using a laboratory spray drier. An experimental design was used for the drying parameters, where the inlet air temperature was varied (160, 180 and 200 °C), feed flow rate (200, 300 and 400 mL/hr), atomization speed (16,000, 20,000 and 24,000 rpm) and outlet air temperature (60, 70 and 80 °C) at different levels. For convenience of experimental design coding was used. The DEDP was collected in a single cyclone separator and was stored after packaging for consecutive 2 months at 25 °C and 4 °C, respectively. The powder yield was calculated from the collected dry mass in the collecting vessel divided by the processed whole egg diluted matter. The total lipids of DEDP samples were determined gravimetrically. The esters of fatty acids in each sample were prepared and analyzed through Gas Chromatograph apparatus. The oxidative stability of DEDP samples was estimated by following standard procedure of peroxide value.

**Results:**

The powder yield of DEDP as a result of different operating conditions was found in the range of 30.06 ± 0.22 g/500 mL to 62.10 ± 0.46 g/500 mL DEs sample. The decreasing trend in moisture content (4.4 ± 0.16% towards 4.0 ± 0.09%) and total fat content (45 ± 0.65 g/100 g towards 41 ± 0.35 g/100 g) in DEDP samples was observed with increased inlet and outlet temperature while fat content increased at high feed flow rate and atomization speed. In this study, loss of PUFAs in DEDP samples was followed due to their active role regarding to human health. For alpha-linolenic (ALA) fatty acids, maximum value at 4 °C observed was 127.32 ± 0.27 mg/50 g egg and 124.43 ± 0.32 mg/50 g egg while the minimum value observed for ALA was 100.15 ± 0.09 mg/50 g egg and 97.15 ± 0.06 mg/50 g egg after 30 and 60 days storage, respectively. The significant decrease trend for eicosapentaenoic (EPA) fatty acids values from 11.78 ± 0.31 mg/50 g egg to 2.18 ± 0.14 mg/50 g egg at 25 °C under spray dried conditions of inlet air temperature (180 °C), feed flow rate (300 mL/hr), atomization speed (24,000 rpm) and outlet air temperature (80 °C) after 60 days storage period was noted. The docosahexaenoic (DHA) fatty acids value in DEDP was decreased from 15.49 ± 0.79 mg/50 g egg (0 day) to 10.10 ± 0.64 mg/50 g egg at 60 days (4 °C) and same decreasing trend was observed at 25 °C. The decreasing order for total omega-3 fatty acids retention in DEDP during storage intervals was found as 162.33 ± 1.64 mg/50 g egg > 158.61 ± 1.53 mg/50 g egg > 148.03 ± 1.57 mg/50 g egg (0, 30 and 60 days stored at 4 °C) and 162.33 ± 1.64 mg/50 g egg > 151.56 ± 1.54 mg/50 g egg > 135.89 ± 1.62 mg/50 g egg (0, 30 and 60 days stored at 25 °C). The peroxide value (PV) levels obtained in DEDP samples after 60 days were higher (0.78 ± 0.06, 0.81 ± 0.02 meq/kg O_2_) when compared to initial readings at 0 day (0.65 ± 0.04 meq/kg O_2_). The PV of DEDP samples reached their maximum peaks after 60 days at 25 °C. The increasing order showed that lipid oxidation increased with storage. However, the overall PV never exceeded the limit of 10 (meq/kg) considered as a threshold limit.

**Conclusions:**

Extreme hot conditions (> 180 °C) of spray dryer reduce the quality of designer egg dry powder. Extreme conditions assist PUFAs loss and decrease in storage stability due to high lipid oxidation.

## Background

Lipids are considered an important component of food as well as most biological systems. Mostly saturated and monounsaturated fatty acids biosynthesized in the body, but polyunsaturated fatty acids (PUFAs) must be provided through the diet or other sources for maintenance of health [1, 2]. PUFAs poses biological and medicinal interest due to multiple beneficial effects on health, including anti-inflammatory, cardioprotective and anticancer activities etc. [3–5]. The designer eggs (DEs) are widely used regarding to human health in providing various essential fatty acids (EFAs) such as omega-3 (or) n-3 fatty acids; Alpha-Linolenic acid (ALA): C_18:3n-3_, Eicosapentaenoic acid (EPA):C_20:5n-3_ and Docosahexaenoic acid (DHA):C_22:6n-3_. DEs show beneficial effects regarding to improve the blood concentration of omega-3 fatty acids and high-density lipoproteins [6].

However, lipid oxidation negatively affects the integrity of biological systems and causes quality deterioration in food. The oxidative instability possesses objectionable off-flavors, loss of nutrients and bioactives that leads to formation of potentially toxic compounds, thus making the lipid or lipid containing foods unsuitable for human health [7]. Destructive irreversible cellular and tissue effects, pathophysiology of numerous diseases and variety of health conditions including inflammation, mutagenesis, atherosclerosis, carcinogenesis and aging process are associated with fatty acid oxidation products in human foods [8–10].

Spray drying is a suspended-particle technology which has a wide range of applications in mostly food, pharmaceutical and biotech industry. In spray drying process, a liquid droplet is rapidly dried, when it comes into contact with a stream of hot air (temperature range from 100 to 300 °C) and convert it into powder form [11]. Spray drying produces powder with good handling, easiness in transportation and highly functional in nature [12–16]. Moreover, dried egg and egg products drive the product manufacture’s attention for ready to use in baked, soups and meat products. The spray dried egg powder has been suggested to be the easily digestible and good source of nutrients from egg products [17]. A comprehensive literature search reported that no significant research has been done on the optimization of the spray drying parameters to produce highest quality designer egg dried powder (DEDP). The present study was undertaken to optimize the spray drying process under different ranges of inlet air temperature, feed flow rate, atomization speed and outlet air temperature to have maximum retention of PUFAs at the different storage periods and temperatures.

## Methods

### Raw materials

The raw materials such as chia seed (*Salvia hispanica L.*) and other cereal grains were procured from grains commercial market, Punjab, Pakistan. The seeds were cleaned to remove any debris or field dirt and any other extraneous matters. The menhaden fish oil was obtained from commercial fish processing industry, Punjab, Pakistan.

### Diet composition and feeding trial

The feeding trial was conducted on medium-heavy Leghorn layers (20 weeks old; uniform weight) in wire-mesh pens of commercial poultry house, Punjab, Pakistan. The birds were used to keep in 17 h light and 7 h dark day. All hens were fed on control diet from 20th week of their age before the trial which was helpful for baseline data. The temperature 25 ± 2 °C and humidity 70 ± 5% remained constant throughout the eight experimental weeks. The Leghorn layers were randomly distributed into control and designer feed treatments of 40 layers each. Each bird activity was observed on daily basis. Routine vaccination and medication were conducted as management suggested. The feed ingredient profile for control and designer eggs production has been presented in Table 1. The mixed crumble feed was produced weekly and packed in air tight feed bins to avoid oxidation and moisture build up and placed in dark cooled room to minimize the exposure to environment. The DEs were produced and collected after 8 weeks of feeding trial.

**Table 1:** Feed ingredient profile for control and designer eggs production

Treatment	Feed Ingredients (g/100 g)^b^												
	Corn	Wheat	Rice polishing	Canola meal	Flaxseed	Chia seed	Fish oil	Gluten (60%)	Soybean meal	Vegetable^a^ oil	Dicalcium phosphate	Lime stone (ground)	Vitamin/mineral premix
Control feed^c^	35	5	15	15	–	–	–	5	8	8	1.5	7	0.5
Designer feed^c^	35	10	8	9	5	10	1	3	8	2	1.5	7	0.5

^a^Cottonseed oil

^b^Crumble form of feed

^c^Isocaloric feeds

### Sample preparation

The DEs (*n* = 600) were cautiously de-shelled and whole egg liquid (*n* = 20) for each treatment was collected in a graduated cylinder. Water was added to the whole egg liquid (60% protein: 40% yolk) and mixed well to make a fine dilution. The concentration of this continuous dilution was 1:1 ratio. Thereafter, homogenization of egg sample was carried out using a homogenizer. A sifting was conducted to eliminate the chalazas and the suspended matter. The whole egg sample was diluted before spray drying to get fine powder [18].

### Spray drying procedure

**Fig. 1:**
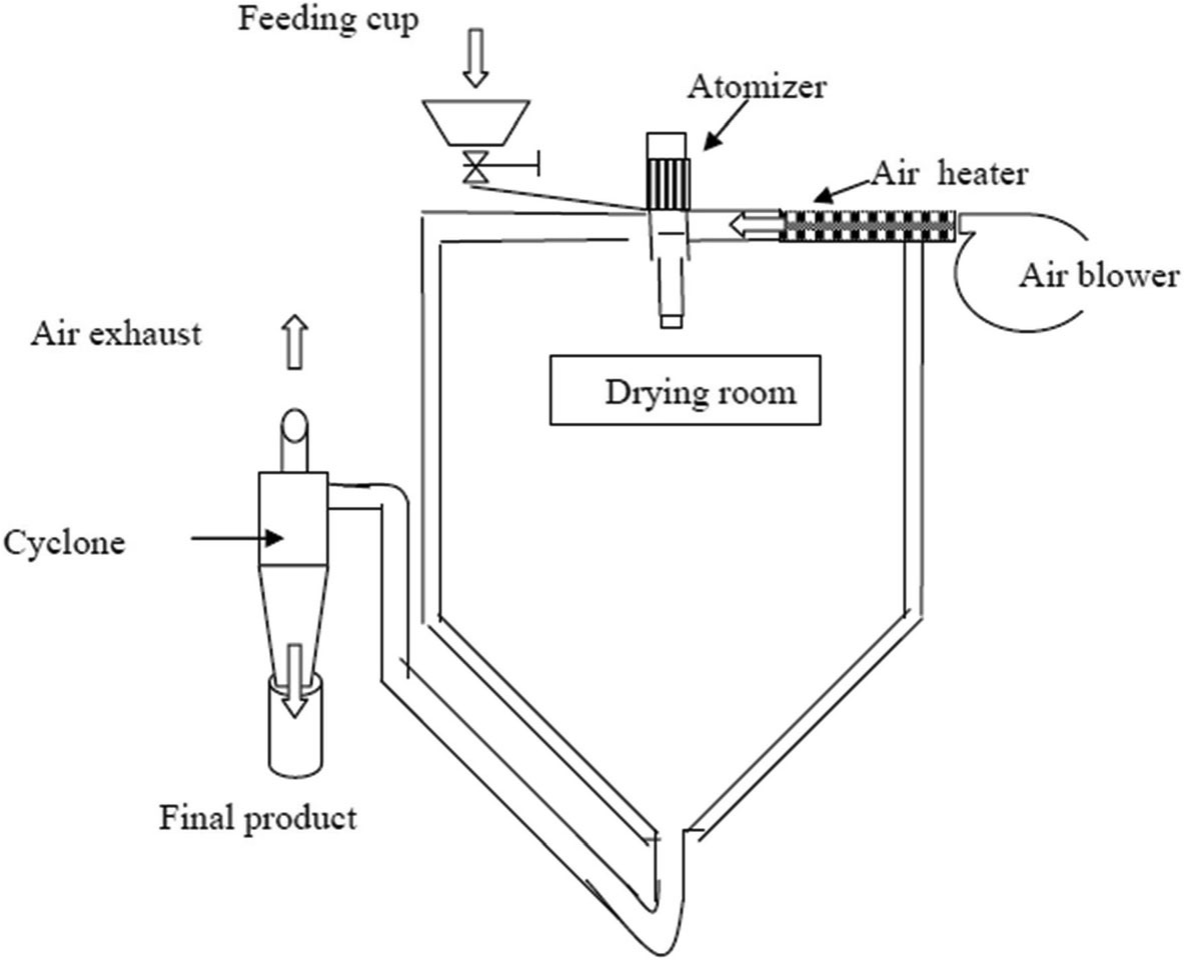
The schematic diagram of the lab-scale spray dryer

A laboratory spray drier No. 1 (Anhydro A/S, Ostmarken 8, DK-2860 Soborg, Copenhagen, Denmark) was used in this study. The schematic diagram of the lab-scale spray dryer is demonstrated in Fig. 1. The internal diameter of representative spray dryer was 1.0 m and 2.6 m were height. The upper cylindrical portion of the unit is 1.3 m in height, and the lower conical section has a height of 1.3 m. The maximum inlet and outlet temperatures are 300 and 90 °C, respectively. Maximum atomizer speed is 50,000 rpm which is obtained by the power supply 0.736 kW electric motor. Air is heated by air heater using a power of 9 kW with compressed air consumption of 120 l/min and compressed air pressure of 4 kg/cm^2^ [19]. An experimental design was used for the drying parameters, where the inlet air temperature was varied (160, 180 and 200 °C), feed flow rate (200, 300 and 400 mL/hr), atomization speed (16,000, 20,000 and 24,000 rpm) and outlet air temperature (60, 70 and 80 °C) at different levels. For convenience of experimental design coding was used which is presented in Table 2. The designer egg dried powder (DEDP) from each treatment (500 mL) was collected in a single cyclone separator and was stored at 25 °C and 4 °C, respectively after packaging for consecutive 2 months.

**Table 2:** Coded and actual levels of independent variables for optimization of response factors as determined by Box-Behnken design

Independent variables	Units	Coded levels		
		–1	0	+ 1
Inlet air temperature	°C	160	180	200
Feed flow rate	mL/hr	200	300	400
Atomization speed	rpm	16,000	20,000	24,000
Outlet air temperature	°C	60	70	80

### Powder yield, total fat and fatty acids composition of DEDP

The powder yield was calculated from the collected dry mass in the collecting vessel divided by the processed whole egg diluted matter. The total lipids of DEDP samples were determined gravimetrically according to the AOAC [20] Method No. 923.07. The esters of fatty acids in each sample were prepared and analyzed through Gas Chromatograph apparatus equipped with an auto sampler, flame-ionization detector (FID) and supelco wax column (30 m × 0.25 μm film coating) according to AOCS [21]. Briefly, transferred 1 g DEDP sample to the screw capped tube (16 X 150 mm). Added 10 mL hexane containing 0.1% BHT (an antioxidant to help prevent the peroxidation of fatty acid containing double bonds). Caped the tube tightly and shaked vigorously for 1 min. Then put in ultrasonic water bath for 5 min. Centrifuged the tube at 1500 X g for 5 min. Put up the experimental tubes into a heating block heated to 60 °C and a stream of nitrogen gas was blown into the tube to facilitate evaporation of the hexane. Toluene (1 mL) was added to 50 mg of sample in a screw top test tube. Subsequently, 2 mL of boron trichloride–methanol solution was added and the mixture was flushed with nitrogen gas for 10 s and heated in a water bath at 60 °C for 10 min. Once cooled, water (2 mL) and hexane (2 mL) were added into the test tube and shaken lightly to extract the fatty acid methyl esters (FAMEs). Anhydrous sodium sulfate was added to the hexane extracts to remove moisture then the anhydrous hexane extracts were transferred into a 10 mL volumetric flask and filled to volume with hexane. The moisture removal step was carried out twice to ensure maximum extraction of FAMEs from the oil. FAMEs were then analyzed by gas chromatography. The FAMEs samples (1 μL) were injected with Helium (1 mL/min) as a carrier gas onto the column, which was programmed for operating conditions such as column oven temperature 160 °C @ 0 min with subsequent increase of 3 °C/min until 180 °C. The column oven temperature was increased from 180 °C to 220 °C @ 1 °C/min and was held for 7.5 min at 220 °C. Split ratio was 50% with injector 240 °C and detector 250 °C temperatures. The peak areas and total fatty acids composition were calculated for each sample by retention time using Varian Chem Station software.

### Peroxide value of DEDP

The peroxide value of DEDP samples was estimated by following the AOCS standard procedure (Method No. Cd 8–53) [21].

### Statistical analysis

The analysis of experiments was carried out according to Montgomery [22]. Each experiment was performed in triplicate and the average values were taken as response. The significance of all terms was analyzed statistically by computing mean square at probability (p) of 0.05 using MATLAB® (Ver. 7.9.0) software (Mathworks, Inc., Natick, USA).

## Results and discussion

**Fig. 2:**
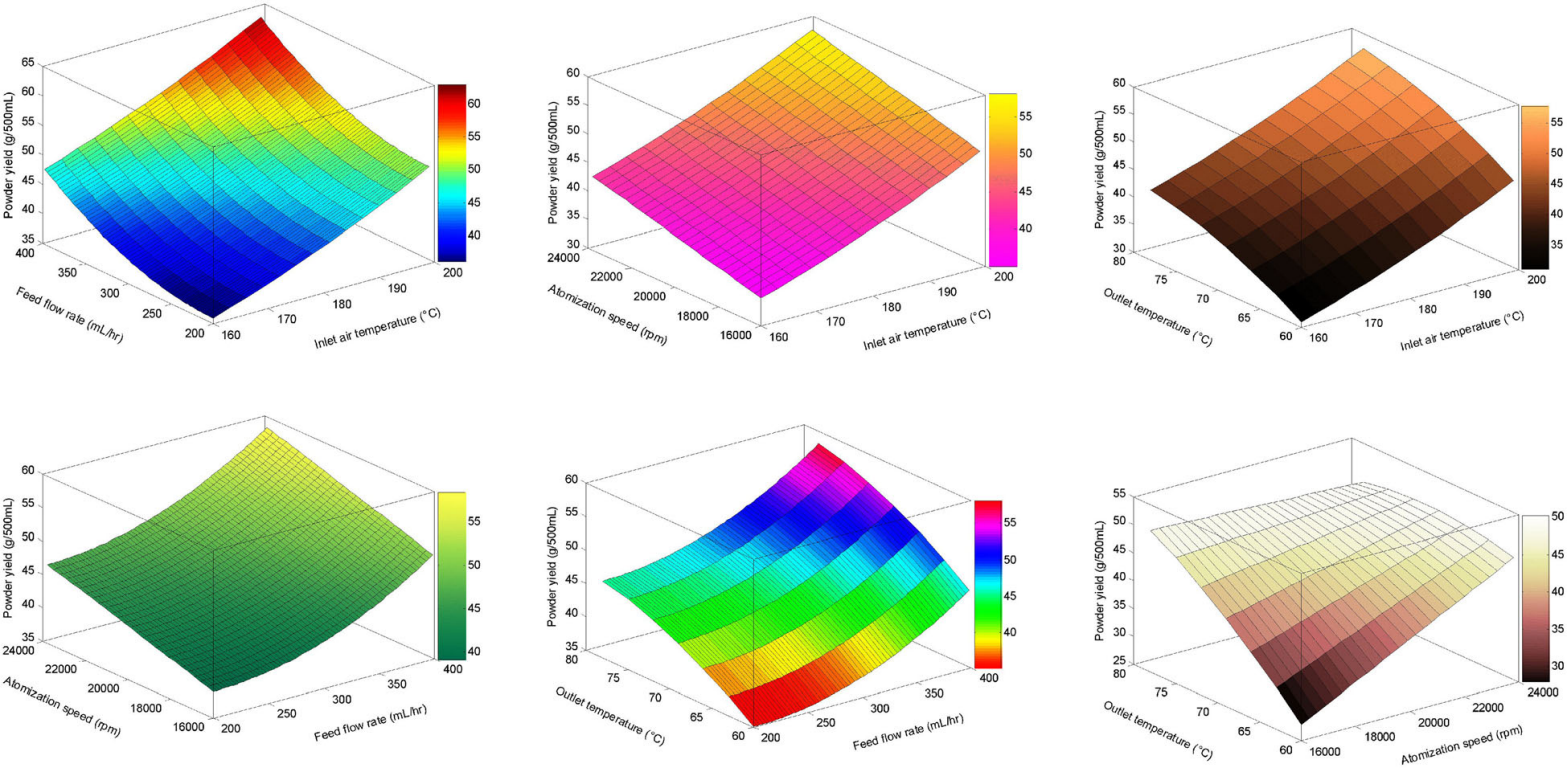
Impact of Spray drying conditions on powder yield in designer egg dried powder

The fatty acids analysis of poultry control and designer feed has been documented in Table 3. In this study, the effects of spray-drying conditions were majorly investigated (Inlet and outlet air temperatures, feed flow rate and atomization pressure) on powder yield along with retention of PUFAs. The powder yield of DEDP as a result of different operating conditions was found in the range of 30.06 ± 0.22 g/500 mL to 62.10 ± 0.46 g/500 mL DEs samples (Fig. 2). The inlet temperature, outlet temperature and the atomization speed were the most major factors affecting the powder yield of DEDP. The results showed that the powder yield decreased with increasing inlet temperature, outlet temperature and the atomization speed. The optimized conditions of inlet air temperature (198–199 °C), feed flow rate (398–399 mL/hr), atomization speed (16000–16,010 rpm) and outlet air temperature (76–77 °C) were found for maximum yield of DEDP samples (66.20 ± 0.20 g/500 mL).

**Table 3:** Fatty acids analysis of poultry control and designer feed

Treatment	Fatty acids (% of TFA^a^)							
	Palmitic	Stearic	Oleic	Linoleic	Linolenic	Arachidonic	Eicosapentaenoic	Docosahexaenoic
Control feed	12.76	15.48	28.33	37.57	2.41	0.5	0.34	0.06
Designer feed	8.33	10.89	23.12	39.45	10.64	0.25	1.44	0.72

^a^Total fatty acids

The spray drying variables caused substantial changes in the whole egg powder yield shown by the previous studies. Same results concluded by Bahnasawy et al. [19] that the powder yield decreased slightly with increasing both atomization speed and drying temperature for all blends under study. These results may be due to production of DEDP with fine particles structure at higher temperature and speed conditions and these conditions force the very fine particles to go out with exhaust air. Atomizer is the heart of spray drying process that disperses material into precise particles so that surface area of the liquid material is increased. In this way, material is dispersed well within the dryer chamber. After atomization, the droplets produced should not be very huge as that condition developed partially dried powder and even nor so tiny in size or structure as that leads to difficulty in recovery of DEDP samples. The final shape and kind of dried powder product depends on the chemical and physical properties of the liquid material, dryer design and operative parameters [11, 23].

The trend in decrease of moisture content was observed with increase in conditions. The decreasing trend in moisture content was found from 4.4 ± 0.16% (highest value) towards 4.0 ± 0.09% (lowest value) in DEDP samples with changes in operating conditions especially inlet and outlet temperature. At the same time increased moisture content was detected at high feed flow rate. The data trend also showed that moisture content was inversely proportional to atomization speed of spray dryer. In a similar way, the total fat content decreased from 45 ± 0.65 g/100 g (highest value) to 41 ± 0.35 g/100 g (lowest value) in DEDP samples with increased inlet and outlet temperature while fat content increased at high feed flow rate and atomization speed.

The normal eggs possessed the ALA (0.78 ± 0.14 mg/50 g egg), EPA (0.11 ± 0.06 mg/50 g egg), DHA (0.14 ± 0.07 mg/50 g egg) and PV (0.324 meq/kg O_2_), respectively. Whereas, the designer eggs before spray drying process contained ALA (130.23 ± 0.28 mg/50 g egg), EPA (15.10 ± 0.37 mg/50 g egg), DHA (20.17 ± 0.67 mg/50 g egg), total omega-3 fatty acids (165.50 ± 2.21 mg/50 g egg) and PV (0.418 meq/kg O_2_), respectively. In this study, loss of PUFAs was followed due to their active role regarding to human health.

To check the reliability of fatty acid retention in DEDP, it was determined in 29 DEDP samples for 30 and 60 storage days at two different temperatures likewise 4 °C and 25 °C, respectively. The results demonstrated that the contents of alpha-linolenic fatty acids were not stable under variable storage intervals at different conditions (Table 4). The inlet air temperature and outlet air temperature were seen to be as major factors affecting the essential fatty acids content in samples. The alpha-linolenic acid, eicosapentaenoic and docosahexaenoic fatty acids contents decreased significantly on storage at higher temperature as compared to lower temperature under different conditions. For alpha-linolenic fatty acids, maximum value at 4 °C observed was 127.32 ± 0.27 mg/50 g egg and 124.43 ± 0.32 mg/50 g egg (spray drying run 22) while the minimum value observed for ALA was 100.15 ± 0.09 mg/50 g egg and 97.15 ± 0.06 mg/50 g egg after 30 and 60 days, respectively. The changes calculated for ALA was 21.98% (at 4 °C after 30 days), 24.32% (at 4 °C after 60 days), 24.01% (at 25 °C after 30 days) and 27.80% (at 25 °C after 60 days), respectively.

**Table 4:** Impact of Spray drying conditions on alpha-linolenic fatty acids retention in designer egg dried powder at different days and storage intervals

Spray dryer process run	Independent variables				ALA (mg/50 g egg)				
	Inlet air temperature (°C)	Feed flow rate (mL/hr)	Atomization speed (rpm)	Outlet temperature (°C)	0 Day	Storage at Temperature 4 °C		Storage at Temperature 25 °C	
						30 Days	60 Days	30 Days	60 Days
1	160 (− 1)	300 (0)	16,000 (−1)	70 (0)	127.57 ± 0.46^ab^	126.22 ± 0.41^b^	123.05 ± 0.44^cd^	123.12 ± 0.42^cd^	118.52 ± 0.41^ef^
2	180 (0)	200 (−1)	20,000 (0)	60 (−1)	118.81 ± 0.70^ef^	117.34 ± 0.62^f^	114.33 ± 0.61^gh^	114.25 ± 0.62^gh^	109.55 ± 0.67^j^
3	180 (0)	300 (0)	16,000 (−1)	80 (+ 1)	116.58 ± 0.47^fg^	117.19 ± 0.44^f^	114.14 ± 0.47^gh^	112.37 ± 0.41^hi^	107.53 ± 0.44^k^
4	180 (0)	400 (+ 1)	20,000 (0)	60 (−1)	125.67 ± 0.54^bc^	124.05 ± 0.44^c^	121.76 ± 0.62^d^	121.53 ± 0.42^d^	116.43 ± 0.31^fg^
5	180 (0)	200 (−1)	16,000 (− 1)	70 (0)	118.30 ± 0.19^ef^	117.04 ± 0.13^f^	114.75 ± 0.15^gh^	114.21 ± 0.14^gh^	109.51 ± 0.16^j^
6	160 (−1)	300 (0)	24,000 (+ 1)	70 (0)	114.75 ± 0.61^gh^	113.66 ± 0.51^h^	110.45 ± 0.34^ij^	110.55 ± 0.43^ij^	105.87 ± 0.75^l^
7(C_1_)	180 (0)	300 (0)	20,000 (0)	70 (0)	114.82 ± 0.70^gh^	113.55 ± 0.45^h^	110.76 ± 0.61^ij^	110.72 ± 0.67^ij^	105.01 ± 0.52^l^
8(C_2_)	180 (0)	300 (0)	20,000 (0)	70 (0)	114.68 ± 0.54^gh^	113.35 ± 0.24^h^	110.41 ± 0.32^ij^	110.42 ± 0.31^ij^	105.42 ± 0.34^l^
9	180 (0)	300 (0)	24,000 (+ 1)	60 (−1)	115.97 ± 0.87^g^	114.72 ± 0.61^gh^	111.95 ± 0.82^i^	111.32 ± 0.24^i^	106.45 ± 0.35^kl^
10	200 (+ 1)	300 (0)	24,000 (+ 1)	70 (0)	101.19 ± 0.12^n^	100.15 ± 0.09^no^	97.15 ± 0.06^p^	97.55 ± 0.08^p^	92.68 ± 0.13^s^
11(C_3_)	180 (0)	300 (0)	20,000 (0)	70 (0)	114.76 ± 0.69^gh^	113.65 ± 0.53^h^	110.22 ± 0.54^ij^	110.01 ± 0.51^ij^	105.03 ± 0.42^l^
12(C_4_)	180 (0)	300 (0)	20,000 (0)	70 (0)	114.75 ± 0.63^gh^	113.64 ± 0.57^h^	110.33 ± 0.64^ij^	110.43 ± 0.37^ij^	105.56 ± 0.46^l^
13	180 (0)	200 (−1)	20,000 (0)	80 (+ 1)	106.03 ± 0.31^kl^	105.44 ± 0.32^l^	102.55 ± 0.40^mn^	102.21 ± 0.22^mn^	97.56 ± 0.42^p^
14	200 (+ 1)	400 (+ 1)	20,000 (0)	70 (0)	111.19 ± 0.49^i^	110.05 ± 0.43^ij^	107.61 ± 0.53^k^	107.31 ± 0.28^k^	102.61 ± 0.52^mn^
15	160 (−1)	400 (+ 1)	20,000 (0)	70 (0)	124.61 ± 0.54^c^	123.55 ± 0.41^cd^	120.75 ± 0.65^de^	120.62 ± 0.56^de^	115.45 ± 0.38^g^
16	180 (0)	400 (+ 1)	20,000 (0)	80 (+ 1)	113.95 ± 0.84^h^	112.65 ± 0.54^hi^	109.74 ± 0.61^j^	109.32 ± 0.57^j^	104.33 ± 0.67^lm^
17	160 (−1)	300 (0)	20,000 (0)	80 (+ 1)	115.57 ± 0.43^g^	114.12 ± 0.35^gh^	111.32 ± 0.28^i^	111.46 ± 0.37^i^	106.78 ± 0.63^kl^
18	200 (+ 1)	300 (0)	20,000 (0)	80 (+ 1)	102.68 ± 0.63^mn^	101.62 ± 0.52^n^	98.33 ± 0.29^op^	98.63 ± 0.50^op^	93.98 ± 0.47^r^
19	160 (−1)	200 (−1)	20,000 (0)	70 (0)	117.87 ± 0.71^f^	116.11 ± 0.64^fg^	113.55 ± 0.42^h^	113.15 ± 0.38^h^	108.09 ± 0.42^jk^
20	180 (0)	400 (+ 1)	16,000 (−1)	70 (0)	125.70 ± 0.62^bc^	124.62 ± 0.51^c^	121.32 ± 0.46^d^	121.43 ± 0.32^d^	116.35 ± 0.27^fg^
21	180 (0)	300 (0)	24,000 (+ 1)	80 (+ 1)	103.92 ± 0.74^m^	102.01 ± 0.61^mn^	99.73 ± 0.66^o^	99.59 ± 0.46^o^	94.67 ± 0.54^qr^
22	180 (0)	300 (0)	16,000 (−1)	60 (− 1)	128.37 ± 0.28^a^	127.32 ± 0.27^ab^	124.43 ± 0.32^c^	124.43 ± 0.31^c^	119.87 ± 0.41^e^
23	180 (0)	400 (+ 1)	24,000 (+ 1)	70 (0)	112.25 ± 0.72^hi^	111.95 ± 0.84^i^	108.11 ± 0.65^jk^	108.55 ± 0.47^jk^	103.34 ± 0.59^m^
24	160 (−1)	300 (0)	20,000 (0)	60 (−1)	127.47 ± 0.38^ab^	126.33 ± 0.24^b^	123.64 ± 0.45^cd^	123.78 ± 0.33^cd^	118.75 ± 0.42^ef^
25(C_5_)	180 (0)	300 (0)	20,000 (0)	70 (0)	114.13 ± 0.20^gh^	113.01 ± 0.24^h^	110.01 ± 0.23^ij^	110.54 ± 0.37^ij^	105.34 ± 0.29^l^
26	200 (+ 1)	300 (0)	16,000 (−1)	70 (0)	114.88 ± 0.51^gh^	113.22 ± 0.44^h^	110.21 ± 0.33^ij^	110.43 ± 0.36^ij^	105.33 ± 0.21^l^
27	180 (0)	200 (−1)	24,000 (+ 1)	70 (0)	105.01 ± 0.22^l^	104.00 ± 0.21^lm^	101.04 ± 0.32^n^	101.34 ± 0.29^n^	96.34 ± 0.23^pq^
28	200 (+ 1)	200 (−1)	20,000 (0)	70 (0)	104.82 ± 0.56^lm^	103.54 ± 0.47^m^	100.23 ± 0.51^no^	100.32 ± 0.57^no^	95.96 ± 0.63^q^
29	200 (+ 1)	300 (0)	20,000 (0)	60 (−1)	114.19 ± 0.12^gh^	113.12 ± 0.15^h^	110.21 ± 0.16^ij^	110.65 ± 0.25^ij^	105.33 ± 0.29^l^

^C1,C2,C3,C4,C5^represent spraying drying process at center points

Experimental model = Box-Behnken design

Total number of spray drying treatments = 29

No of replicates = 03

^a-s^values with similar letters show homogenous group within row and column (*p* > 0.05)

The trend for effects of various storage time intervals for eicosapentaenoic fatty acids is shown in Table 5. The minimum value for EPA observed was 10.02 ± 0.21 mg/50 g egg under four factors of spray drier i.e. inlet air temperature (200 °C), feed flow rate (300 mL/hr), atomization speed (20,000 rpm), outlet air temperature (80 °C) stored at 4 °C after 30 days of storage. The EPA trend showed that significant decrease 11.78 ± 0.31 mg/50 g egg to 2.18 ± 0.14 mg/50 g egg at 25 °C under spray drier factors inlet air temperature (180 °C), feed flow rate (300 mL/hr), atomization speed (24,000 rpm) and outlet air temperature (80 °C) after 60 days storage period. The EPA changes were 31.13% (at 4 °C after 30 days), 61.64% (at 4 °C after 60 days), 51.89% (at 25 °C after 30 days) and 85.01% (at 25 °C after 60 days), respectively.

**Table 5:** Impact of Spray drying conditions on eicosapentaenoic fatty acids retention in designer egg dried powder at different days and storage intervals

Spray dryer process run	Independent variables				EPA (mg/50 g egg)				
	Inlet air temperature (°C)	Feed flow rate (mL/hr)	Atomization speed (rpm)	Outlet temperature (°C)	0 Day	Storage at Temperature 4 °C		Storage at Temperature 25 °C	
						30 Days	60 Days	30 Days	60 Days
1	160 (−1)	300 (0)	16,000 (− 1)	70 (0)	14.51 ± 0.46^a^	13.22 ± 0.41^ab^	9.76 ± 0.41^cd^	10.92 ± 0.41^c^	5.14 ± 0.44^ef^
2	180 (0)	200 (−1)	20,000 (0)	60 (−1)	13.55 ± 0.38^ab^	12.15 ± 0.35^b^	8.81 ± 0.35^d^	9.91 ± 0.35^cd^	4.75 ± 0.32^f^
3	180 (0)	300 (0)	16,000 (−1)	80 (+ 1)	13.22 ± 0.34^ab^	12.11 ± 0.32^b^	8.40 ± 0.32^d^	9.84 ± 0.32^cd^	4.72 ± 0.31^f^
4	180 (0)	400 (+ 1)	20,000 (0)	60 (−1)	14.25 ± 0.41^a^	13.05 ± 0.40^ab^	9.42 ± 0.40^cd^	10.89 ± 0.40^c^	5.69 ± 0.34^ef^
5	180 (0)	200 (−1)	16,000 (− 1)	70 (0)	13.45 ± 0.39^ab^	12.19 ± 0.35^b^	5.58 ± 0.35^ef^	9.86 ± 0.35^cd^	4.88 ± 0.32^f^
6	160 (−1)	300 (0)	24,000 (+ 1)	70 (0)	13.05 ± 0.33^ab^	12.00 ± 0.31^b^	8.82 ± 0.31^d^	9.00 ± 0.31^cd^	4.75 ± 0.29^f^
7(C_1_)	180 (0)	300 (0)	20,000 (0)	70 (0)	13.09 ± 0.34^ab^	12.03 ± 0.32^b^	8.86 ± 0.32^d^	9.74 ± 0.32^cd^	4.65 ± 0.30^f^
8(C_2_)	180 (0)	300 (0)	20,000 (0)	70 (0)	13.10 ± 0.38^ab^	12.15 ± 0.37^b^	8.87 ± 0.37^d^	9.72 ± 0.37^cd^	4.61 ± 0.31^f^
9	180 (0)	300 (0)	24,000 (+ 1)	60 (−1)	13.05 ± 0.39^ab^	12.11 ± 0.35^b^	8.83 ± 0.35^d^	9.82 ± 0.35^cd^	4.67 ± 0.38^f^
10	200 (+ 1)	300 (0)	24,000 (+ 1)	70 (0)	11.53 ± 0.29^bc^	10.05 ± 0.21^c^	6.90 ± 0.21^e^	7.99 ± 0.21^de^	2.91 ± 0.27^g^
11(C_3_)	180 (0)	300 (0)	20,000 (0)	70 (0)	13.11 ± 0.33^ab^	12.18 ± 0.30^b^	8.81 ± 0.30^d^	9.91 ± 0.30^cd^	4.62 ± 0.29^f^
12(C_4_)	180 (0)	300 (0)	20,000 (0)	70 (0)	13.12 ± 0.38^ab^	12.11 ± 0.33^b^	8.82 ± 0.33^d^	9.92 ± 0.33^cd^	4.63 ± 0.31^f^
13	180 (0)	200 (−1)	20,000 (0)	80 (+ 1)	12.15 ± 0.28^b^	11.09 ± 0.22^bc^	7.11 ± 0.22^de^	8.00 ± 0.22^d^	3.89 ± 0.18^fg^
14	200 (+ 1)	400 (+ 1)	20,000 (0)	70 (0)	12.72 ± 0.26^b^	11.18 ± 0.20^bc^	7.71 ± 0.20^de^	8.82 ± 0.20^d^	3.35 ± 0.23^fg^
15	160 (−1)	400 (+ 1)	20,000 (0)	70 (0)	14.25 ± 0.45^a^	13.03 ± 0.41^ab^	9.15 ± 0.41^cd^	10.45 ± 0.41^c^	5.88 ± 0.28^ef^
16	180 (0)	400 (+ 1)	20,000 (0)	80 (+ 1)	12.93 ± 0.29^b^	11.53 ± 0.21^bc^	7.35 ± 0.21^de^	8.83 ± 0.21^d^	3.37 ± 0.32^fg^
17	160 (−1)	300 (0)	20,000 (0)	80 (+ 1)	13.24 ± 0.35^ab^	12.06 ± 0.33^b^	8.34 ± 0.33^d^	9.91 ± 0.33^cd^	4.67 ± 0.39^f^
18	200 (+ 1)	300 (0)	20,000 (0)	80 (+ 1)	11.75 ± 0.27^bc^	10.02 ± 0.21^c^	6.98 ± 0.21^e^	7.11 ± 0.21^de^	2.29 ± 0.15^g^
19	160 (−1)	200 (−1)	20,000 (0)	70 (0)	13.45 ± 0.39^ab^	12.19 ± 0.31^b^	8.31 ± 0.31^d^	9.91 ± 0.31^cd^	4.22 ± 0.21^f^
20	180 (0)	400 (+ 1)	16,000 (−1)	70 (0)	14.26 ± 0.42^a^	13.10 ± 0.40^ab^	9.34 ± 0.40^cd^	10.13 ± 0.40^c^	5.67 ± 0.23^ef^
21	180 (0)	300 (0)	24,000 (+ 1)	80 (+ 1)	11.78 ± 0.31^bc^	10.35 ± 0.28^c^	6.70 ± 0.28^e^	7.00 ± 0.28^de^	2.18 ± 0.14^g^
22	180 (0)	300 (0)	16,000 (−1)	60 (− 1)	14.55 ± 0.46^a^	13.10 ± 0.37^ab^	9.59 ± 0.37^cd^	10.33 ± 0.37^c^	5.91 ± 0.27^ef^
23	180 (0)	400 (+ 1)	24,000 (+ 1)	70 (0)	12.72 ± 0.34^b^	11.31 ± 0.32^bc^	7.93 ± 0.32^de^	8.82 ± 0.32^d^	3.89 ± 0.30^fg^
24	160 (− 1)	300 (0)	20,000 (0)	60 (−1)	14.55 ± 0.41^a^	13.13 ± 0.38^ab^	9.83 ± 0.38^cd^	10.81 ± 0.38^c^	5.86 ± 0.25^ef^
25(C_5_)	180 (0)	300 (0)	20,000 (0)	70 (0)	13.08 ± 0.33^ab^	12.14 ± 0.30^b^	8.85 ± 0.30^d^	9.71 ± 0.30^cd^	4.51 ± 0.21^f^
26	200 (+ 1)	300 (0)	16,000 (− 1)	70 (0)	13.05 ± 0.37^ab^	12.31 ± 0.31^b^	8.01 ± 0.31^d^	9.09 ± 0.31^cd^	4.42 ± 0.28^f^
27	180 (0)	200 (−1)	24,000 (+ 1)	70 (0)	11.95 ± 0.29^bc^	10.72 ± 0.22^c^	6.02 ± 0.22^e^	7.22 ± 0.22^de^	2.39 ± 0.11^g^
28	200 (+ 1)	200 (−1)	20,000 (0)	70 (0)	11.94 ± 0.32^bc^	10.70 ± 0.29^c^	6.00 ± 0.29^e^	7.73 ± 0.29^de^	2.26 ± 0.19^g^
29	200 (+ 1)	300 (0)	20,000 (0)	60 (−1)	13.05 ± 0.36^ab^	12.10 ± 0.31^b^	8.11 ± 0.31^d^	9.71 ± 0.31^cd^	4.54 ± 0.36^f^

^C1,C2,C3,C4,C5^represent spraying drying process at center points

Experimental model = Box-Behnken design

Total number of spray drying treatments = 29

No of replicates = 03

^a-g^values with similar letters show homogenous group within row and column (*p* > 0.05)

The minimum values for DEDP samples regarding to retention of docosahexaenoic fatty acids stored at two different storage temperatures 4 °C and 25 °C observed at the same spray drier conditions (0, 30 and 60 days) as shown in Table 6. The DHA value in DEDP was decreased from 15.49 ± 0.79 mg/50 g egg (0 day) to 10.10 ± 0.64 mg/50 g egg at 60 days (4 °C) and same decreasing trend was observed at 25 °C. The trend in percent changes calculated for EPA was 8.26% (at 4 °C after 30 days), 34.79% (at 4 °C after 60 days), 27.88% (at 25 °C after 30 days) and 61.20% (at 25 °C after 60 days), respectively. The decreasing order for total omega-3 fatty acids retention in DEDP obtained by keeping spray drier factors (i.e. inlet air temperature and feed flow rate at medium level whereas atomization and outlet air temperature at minimum level) during storage intervals was found 162.33 ± 1.64 mg/50 g egg > 158.61 ± 1.53 mg/50 g egg > 148.03 ± 1.57 mg/50 g egg (0, 30 and 60 days stored at 4 °C) and 162.33 ± 1.64 mg/50 g egg > 151.56 ± 1.54 mg/50 g egg > 135.89 ± 1.62 mg/50 g egg (0, 30 and 60 days stored at 25 °C) (Table 7).

**Table 6:** Impact of Spray drying conditions on docosahexaenoic fatty acids retention in designer egg dried powder at different days and storage intervals

Spray dryer process run	Independent variables				DHA (mg/50 g egg)				
	Inlet air temperature (°C)	Feed flow rate (mL/hr)	Atomization speed (rpm)	Outlet temperature (°C)	0 Day	Storage at Temperature 4 °C		Storage at Temperature 25 °C	
						30 Days	60 Days	30 Days	60 Days
1	160 (− 1)	300 (0)	16,000 (− 1)	70 (0)	19.46 ± 0.88^a^	18.21 ± 0.81^ab^	14.15 ± 0.79^cd^	15.15 ± 0.79^c^	10.45 ± 0.73^ef^
2	180 (0)	200 (−1)	20,000 (0)	60 (− 1)	18.07 ± 0.85^ab^	17.85 ± 0.71^b^	13.65 ± 0.70^d^	14.65 ± 0.70^cd^	9.63 ± 0.71^f^
3	180 (0)	300 (0)	16,000 (−1)	80 (+ 1)	17.62 ± 0.79^b^	16.40 ± 0.74^bc^	12.35 ± 0.71^de^	13.55 ± 0.71^d^	8.55 ± 0.67^fg^
4	180 (0)	400 (+ 1)	20,000 (0)	60 (−1)	19.32 ± 0.72^a^	18.20 ± 0.69^ab^	14.11 ± 0.63^cd^	15.22 ± 0.63^c^	10.76 ± 0.64^ef^
5	180 (0)	200 (−1)	16,000 (− 1)	70 (0)	18.45 ± 0.73^ab^	17.21 ± 0.68^b^	13.18 ± 0.61^d^	14.00 ± 0.61^cd^	9.67 ± 0.65^f^
6	160 (− 1)	300 (0)	24,000 (+ 1)	70 (0)	17.42 ± 0.81^b^	16.12 ± 0.78^bc^	12.02 ± 0.75^de^	13.51 ± 0.75^d^	8.45 ± 0.71^fg^
7(C_1_)	180 (0)	300 (0)	20,000 (0)	70 (0)	17.44 ± 0.80^b^	16.11 ± 0.71^bc^	12.01 ± 0.70^de^	13.59 ± 0.70^d^	8.41 ± 0.63^fg^
8(C_2_)	180 (0)	300 (0)	20,000 (0)	70 (0)	17.46 ± 0.78^b^	16.13 ± 0.72^bc^	12.03 ± 0.71^de^	13.58 ± 0.71^d^	8.66 ± 0.71^fg^
9	180 (0)	300 (0)	24,000 (+ 1)	60 (−1)	17.48 ± 0.82^b^	16.18 ± 0.74^bc^	12.09 ± 0.75^de^	13.52 ± 0.75^d^	8.72 ± 0.72^fg^
10	200 (+ 1)	300 (0)	24,000 (+ 1)	70 (0)	15.49 ± 0.79^c^	14.21 ± 0.69^cd^	10.10 ± 0.64^ef^	11.17 ± 0.64^e^	6.01 ± 0.63^h^
11(C_3_)	180 (0)	300 (0)	20,000 (0)	70 (0)	17.41 ± 0.83^b^	16.15 ± 0.81^bc^	12.04 ± 0.88^de^	13.49 ± 0.88^d^	8.58 ± 0.81^fg^
12(C_4_)	180 (0)	300 (0)	20,000 (0)	70 (0)	17.43 ± 0.77^b^	16.19 ± 0.70^bc^	12.11 ± 0.73^de^	13.33 ± 0.73^d^	8.49 ± 0.73^fg^
13	180 (0)	200 (−1)	20,000 (0)	80 (+ 1)	16.27 ± 0.84^bc^	15.00 ± 0.72^c^	11.85 ± 0.81^e^	12.89 ± 0.81^de^	7.54 ± 0.82^g^
14	200 (+ 1)	400 (+ 1)	20,000 (0)	70 (0)	17.08 ± 0.76^b^	16.22 ± 0.71^bc^	12.66 ± 0.73^de^	13.77 ± 0.73^d^	8.09 ± 0.71^fg^
15	160 (−1)	400 (+ 1)	20,000 (0)	70 (0)	19.11 ± 0.72^a^	18.35 ± 0.70^ab^	14.63 ± 0.71^cd^	15.79 ± 0.71^c^	10.00 ± 0.72^ef^
16	180 (0)	400 (+ 1)	20,000 (0)	80 (+ 1)	17.23 ± 0.85^b^	16.01 ± 0.88^bc^	12.89 ± 0.83^de^	13.91 ± 0.83^d^	8.55 ± 0.83^fg^
17	160 (−1)	300 (0)	20,000 (0)	80 (+ 1)	17.65 ± 0.78^b^	16.39 ± 0.72^bc^	12.21 ± 0.76^de^	13.41 ± 0.76^d^	8.68 ± 0.75^fg^
18	200 (+ 1)	300 (0)	20,000 (0)	80 (+ 1)	15.62 ± 0.86^c^	14.39 ± 0.82^cd^	10.23 ± 0.85^ef^	11.81 ± 0.85^e^	6.58 ± 0.84^h^
19	160 (−1)	200 (−1)	20,000 (0)	70 (0)	18.10 ± 0.89^ab^	17.90 ± 0.83^b^	13.59 ± 0.84^d^	14.73 ± 0.84^cd^	9.55 ± 0.82^f^
20	180 (0)	400 (+ 1)	16,000 (−1)	70 (0)	19.03 ± 0.91^a^	18.79 ± 0.93^ab^	14.61 ± 0.92^cd^	15.69 ± 0.92^c^	10.80 ± 0.91^ef^
21	180 (0)	300 (0)	24,000 (+ 1)	80 (+ 1)	15.62 ± 0.69^c^	14.33 ± 0.63^cd^	10.11 ± 0.65^ef^	11.21 ± 0.65^e^	6.78 ± 0.65^h^
22	180 (0)	300 (0)	16,000 (−1)	60 (−1)	19.41 ± 0.92^a^	18.19 ± 0.96^ab^	14.01 ± 0.93^cd^	15.31 ± 0.93^c^	10.11 ± 0.89^ef^
23	180 (0)	400 (+ 1)	24,000 (+ 1)	70 (0)	17.02 ± 0.75^b^	16.60 ± 0.71^bc^	12.49 ± 0.72^de^	13.69 ± 0.72^d^	8.41 ± 0.74^fg^
24	160 (−1)	300 (0)	20,000 (0)	60 (−1)	19.43 ± 0.88^a^	18.40 ± 0.82^ab^	14.23 ± 0.85^cd^	15.99 ± 0.85^c^	10.21 ± 0.85^ef^
25(C_5_)	180 (0)	300 (0)	20,000 (0)	70 (0)	17.40 ± 0.72^b^	16.22 ± 0.76^bc^	12.09 ± 0.79^de^	13.40 ± 0.79^d^	8.55 ± 0.75^fg^
26	200 (+ 1)	300 (0)	16,000 (−1)	70 (0)	17.45 ± 0.82^b^	16.25 ± 0.79^bc^	12.10 ± 0.80^de^	13.05 ± 0.80^d^	8.59 ± 0.84^fg^
27	180 (0)	200 (−1)	24,000 (+ 1)	70 (0)	16.04 ± 0.77^bc^	15.30 ± 0.71^c^	11.63 ± 0.75^e^	12.91 ± 0.75^de^	7.98 ± 0.73^g^
28	200 (+ 1)	200 (−1)	20,000 (0)	70 (0)	16.09 ± 0.79^bc^	15.30 ± 0.74^c^	11.51 ± 0.76^e^	12.72 ± 0.76^de^	7.94 ± 0.72^g^
29	200 (+ 1)	300 (0)	20,000 (0)	60 (−1)	17.46 ± 0.83^b^	16.22 ± 0.81^bc^	12.00 ± 0.82^de^	13.20 ± 0.82^d^	8.12 ± 0.82^fg^

^C1,C2,C3,C4,C5^represent spraying drying process at center points

Experimental model = Box-Behnken design

Total number of spray drying treatments = 29

No of replicates = 03

^a-h^values with similar letters show homogenous group within row and column (*p* > 0.05)

**Table 7:** Impact of Spray drying conditions on total omega-3 fatty acids retention in designer egg dried powder at different days and storage intervals

Spray dryer process run	Independent variables				Total omega-3 fatty acids (mg/50 g egg)				
	Inlet air temperature (°C)	Feed flow rate (mL/hr)	Atomization speed (rpm)	Outlet temperature (°C)	0 Day	Storage at Temperature 4 °C		Storage at Temperature 25 °C	
						30 Days	60 Days	30 Days	60 Days
1	160 (− 1)	300 (0)	16,000 (− 1)	70 (0)	161.54 ± 2.15^ab^	157.65 ± 2.21^c^	146.96 ± 2.28^gh^	149.19 ± 2.14^f^	134.11 ± 2.11^m^
2	180 (0)	200 (−1)	20,000 (0)	60 (−1)	150.43 ± 2.14^ef^	147.34 ± 2.22^g^	136.79 ± 2.07^l^	138.81 ± 2.01^k^	123.93 ± 2.02^r^
3	180 (0)	300 (0)	16,000 (−1)	80 (+ 1)	147.40 ± 2.47^g^	145.7 ± 2.33^h^	134.89 ± 2.12^m^	135.76 ± 1.80^lm^	120.08 ± 1.93st
4	180 (0)	400 (+ 1)	20,000 (0)	60 (−1)	159.34 ± 1.88^b^	155.3 ± 1.84^d^	145.29 ± 1.91^h^	147.64 ± 1.65^g^	132.88 ± 1.44^n^
5	180 (0)	200 (−1)	16,000 (− 1)	70 (0)	150.20 ± 2.11^ef^	146.44 ± 1.94^gh^	133.51 ± 1.72^mn^	138.07 ± 1.76^k^	124.06 ± 1.95^qr^
6	160 (−1)	300 (0)	24,000 (+ 1)	70 (0)	145.22 ± 1.88^h^	141.78 ± 1.53^ij^	131.29 ± 1.61^no^	133.06 ± 1.57^mn^	119.07 ± 1.44^t^
7(C_1_)	180 (0)	300 (0)	20,000 (0)	70 (0)	145.35 ± 1.52^h^	141.69 ± 1.88^ij^	131.63 ± 1.85^no^	134.75 ± 1.82^m^	118.77 ± 1.98^tu^
8(C_2_)	180 (0)	300 (0)	20,000 (0)	70 (0)	145.24 ± 1.57^h^	141.52 ± 1.85^ij^	131.31 ± 1.96^no^	134.72 ± 1.74^m^	118.85 ± 1.96^tu^
9	180 (0)	300 (0)	24,000 (+ 1)	60 (−1)	146.5 ± 1.78^gh^	142.91 ± 1.84^i^	132.87 ± 1.81^n^	134.16 ± 1.65^m^	119.84 ± 1.74^t^
10	200 (+ 1)	300 (0)	24,000 (+ 1)	70 (0)	128.21 ± 1.55^p^	124.41 ± 1.15^qr^	114.16 ± 1.32^vw^	116.71 ± 1.46^uv^	101.60 ± 1.65^y^
11(C_3_)	180 (0)	300 (0)	20,000 (0)	70 (0)	145.28 ± 1.69^h^	141.78 ± 1.58^ij^	131.46 ± 1.75^no^	134.81 ± 1.84^m^	118.63 ± 1.78^tu^
12(C_4_)	180 (0)	300 (0)	20,000 (0)	70 (0)	145.30 ± 1.63^h^	141.81 ± 1.52^ij^	131.56 ± 1.89^no^	134.68 ± 1.62^m^	118.78 ± 1.68^tu^
13	180 (0)	200 (−1)	20,000 (0)	80 (+ 1)	134.38 ± 1.81^m^	131.53 ± 1.61^no^	121.51 ± 1.72^s^	123.1 ± 1.74^r^	108.99 ± 1.83^w^
14	200 (+ 1)	400 (+ 1)	20,000 (0)	70 (0)	140.97 ± 1.95^j^	137.45 ± 1.84^kl^	127.98 ± 1.81^pq^	129.9 ± 1.96^op^	114.05 ± 1.75^vw^
15	160 (−1)	400 (+ 1)	20,000 (0)	70 (0)	157.92 ± 1.74^c^	154.93 ± 1.65^de^	144.53 ± 1.62^hi^	146.86 ± 1.56^gh^	131.33 ± 1.55^no^
16	180 (0)	400 (+ 1)	20,000 (0)	80 (+ 1)	154.08 ± 1.61^de^	140.19 ± 1.57^j^	129.98 ± 1.54^op^	132.06 ± 1.56^n^	116.25 ± 1.57^uv^
17	160 (− 1)	300 (0)	20,000 (0)	80 (+ 1)	146.42 ± 1.84^gh^	142.57 ± 1.75^i^	131.85 ± 1.87^no^	134.78 ± 1.71^m^	120.13 ± 1.76st
18	200 (+ 1)	300 (0)	20,000 (0)	80 (+ 1)	130.0 ± 1.34^o^	126.03 ± 1.23^q^	115.54 ± 1.26^v^	117.55 ± 1.21^u^	102.85 ± 1.32^xy^
19	160 (− 1)	200 (− 1)	20,000 (0)	70 (0)	149.42 ± 1.55^f^	146.2 ± 1.44^gh^	135.49 ± 1.48^lm^	137.79 ± 1.58^kl^	121.86 ± 1.41^s^
20	180 (0)	400 (+ 1)	16,000 (−1)	70 (0)	158.93 ± 1.72^bc^	156.51 ± 1.61^cd^	145.27 ± 1.85^h^	147.25 ± 1.74^g^	132.82 ± 1.68^n^
21	180 (0)	300 (0)	24,000 (+ 1)	80 (+ 1)	131.24 ± 1.91^no^	126.69 ± 1.82^q^	116.54 ± 1.64^uv^	117.8 ± 1.88^u^	103.63 ± 1.77^x^
22	180 (0)	300 (0)	16,000 (−1)	60 (−1)	162.33 ± 1.64^a^	158.61 ± 1.53^bc^	148.03 ± 1.57^fg^	151.56 ± 1.54^e^	135.89 ± 1.62^lm^
23	180 (0)	400 (+ 1)	24,000 (+ 1)	70 (0)	141.97 ± 1.46^ij^	139.86 ± 1.45^jk^	128.53 ± 1.46^p^	131.54 ± 1.53^no^	115.64 ± 1.32^v^
24	160 (−1)	300 (0)	20,000 (0)	60 (−1)	161.45 ± 1.51^ab^	157.86 ± 1.42^c^	147.7 ± 1.55^g^	150.81 ± 1.48^ef^	134.82 ± 1.37^m^
25(C_5_)	180 (0)	300 (0)	20,000 (0)	70 (0)	145.45 ± 1.42^h^	141.67 ± 1.81^ij^	131.55 ± 1.72^no^	134.55 ± 1.93^m^	118.95 ± 1.58^tu^
26	200 (+ 1)	300 (0)	16,000 (−1)	70 (0)	145.38 ± 1.45^h^	141.78 ± 1.44^ij^	130.32 ± 1.33^o^	132.57 ± 1.32^n^	118.34 ± 1.41^tu^
27	180 (0)	200 (−1)	24,000 (+ 1)	70 (0)	133.00 ± 1.32^mn^	130.02 ± 1.21^o^	118.69 ± 1.22^tu^	122.1 ± 1.34^rs^	106.71 ± 1.28^wx^
28	200 (+ 1)	200 (− 1)	20,000 (0)	70 (0)	132.85 ± 1.25^n^	129.54 ± 1.24^op^	117.74 ± 1.28^u^	121.01 ± 1.22^s^	106.16 ± 1.31^wx^
29	200 (+ 1)	300 (0)	20,000 (0)	60 (−1)	144.7 ± 1.44^hi^	141.44 ± 1.32^ij^	130.32 ± 1.44^o^	133.58 ± 1.28^mn^	117.99 ± 1.33^u^

^C1,C2,C3,C4,C5^represent spraying drying process at center points

Experimental model = Box-Behnken design

Total number of spray drying treatments = 29

No of replicates = 03

^a-y^values with similar letters show homogenous group within row and column (p > 0.05)

The effects of various spray drier conditions and storage on peroxide value in DEDP have been shown in Table 8. The PV of DEDP samples reached their maximum peaks after 60 days at 25 °C. The increasing order shows that lipid oxidation increased with storage. The peroxides are considered as early oxidation products with relatively short induction periods during which they form, accumulate and dissipate. It seems true that the DEDP samples stored for 30 days at lower temperature were relatively stable than stored at higher temperature for 60 days. The overall PV never exceeded the limit of 10 (meq/kg) considered as a threshold limit. The PV levels obtained from 60 days in DEDP samples were higher (0.78 ± 0.06, 0.81 ± 0.02 meq/kg O_2_) when compared to initial readings 0 day (0.65 ± 0.04 meq/kg O_2_).

**Table 8:** Impact of spray drying conditions on peroxide value in designer egg dried powder at different days and storage intervals

Spray dryer process run	Independent variables				Peroxide value (meq/kg O_2_)				
	Inlet air temperature (°C)	Feed flow rate (mL/hr)	Atomization speed (rpm)	Outlet temperature (°C)	0 Day	Storage at Temperature 4 °C		Storage at Temperature 25 °C	
						30 Days	60 Days	30 Days	60 Days
1	160 (− 1)	300 (0)	16,000 (− 1)	70 (0)	0.46 ± 0.05^pq^	0.52 ± 0.01^n^	0.59 ± 0.08^jk^	0.53 ± 0.04^mn^	0.62 ± 0.01^i^
2	180 (0)	200 (− 1)	20,000 (0)	60 (−1)	0.45 ± 0.04^q^	0.51 ± 0.02^no^	0.58 ± 0.07^k^	0.52 ± 0.01^n^	0.61 ± 0.02^ij^
3	180 (0)	300 (0)	16,000 (−1)	80 (+ 1)	0.58 ± 0.07^k^	0.64 ± 0.03^h^	0.71 ± 0.02^de^	0.65 ± 0.02^gh^	0.74 ± 0.03^c^
4	180 (0)	400 (+ 1)	20,000 (0)	60 (−1)	0.49 ± 0.08^op^	0.56 ± 0.04^l^	0.62 ± 0.01^i^	0.58 ± 0.05^k^	0.65 ± 0.04^gh^
5	180 (0)	200 (−1)	16,000 (− 1)	70 (0)	0.50 ± 0.01^o^	0.56 ± 0.05^l^	0.63 ± 0.02^hi^	0.57 ± 0.06^kl^	0.67 ± 0.05^fg^
6	160 (−1)	300 (0)	24,000 (+ 1)	70 (0)	0.51 ± 0.02^no^	0.55 ± 0.03^lm^	0.62 ± 0.01^i^	0.58 ± 0.07^k^	0.68 ± 0.04^f^
7(C_1_)	180 (0)	300 (0)	20,000 (0)	70 (0)	0.53 ± 0.02^mn^	0.59 ± 0.08^jk^	0.66 ± 0.05^g^	0.60 ± 0.05^j^	0.69 ± 0.08^ef^
8(C_2_)	180 (0)	300 (0)	20,000 (0)	70 (0)	0.53 ± 0.04^mn^	0.59 ± 0.05^jk^	0.66 ± 0.06^g^	0.60 ± 0.04^j^	0.69 ± 0.06^ef^
9	180 (0)	300 (0)	24,000 (+ 1)	60 (−1)	0.49 ± 0.08^op^	0.55 ± 0.04^lm^	0.62 ± 0.01^i^	0.56 ± 0.05^l^	0.65 ± 0.04^gh^
10	200 (+ 1)	300 (0)	24,000 (+ 1)	70 (0)	0.60 ± 0.05^j^	0.66 ± 0.05^g^	0.73 ± 0.02^cd^	0.67 ± 0.06^fg^	0.76 ± 0.05^b^
11(C_3_)	180 (0)	300 (0)	20,000 (0)	70 (0)	0.53 ± 0.09^mn^	0.59 ± 0.07^jk^	0.66 ± 0.04^g^	0.60 ± 0.03^j^	0.69 ± 0.07^ef^
12(C_4_)	180 (0)	300 (0)	20,000 (0)	70 (0)	0.53 ± 0.03^mn^	0.59 ± 0.02^jk^	0.66 ± 0.07^g^	0.60 ± 0.02^j^	0.69 ± 0.05^ef^
13	180 (0)	200 (−1)	20,000 (0)	80 (+ 1)	0.58 ± 0.01^k^	0.64 ± 0.01^h^	0.71 ± 0.02^de^	0.65 ± 0.04^gh^	0.75 ± 0.03^bc^
14	200 (+ 1)	400 (+ 1)	20,000 (0)	70 (0)	0.60 ± 0.05^j^	0.65 ± 0.04^gh^	0.72 ± 0.01^d^	0.67 ± 0.06^fg^	0.76 ± 0.05^b^
15	160 (−1)	400 (+ 1)	20,000 (0)	70 (0)	0.50 ± 0.04^o^	0.56 ± 0.05^l^	0.63 ± 0.02^hi^	0.57 ± 0.06^kl^	0.66 ± 0.05^g^
16	180 (0)	400 (+ 1)	20,000 (0)	80 (+ 1)	0.62 ± 0.01^i^	0.68 ± 0.07^f^	0.75 ± 0.04^bc^	0.67 ± 0.06^fg^	0.78 ± 0.07^ab^
17	160 (−1)	300 (0)	20,000 (0)	80 (+ 1)	0.55 ± 0.04^lm^	0.61 ± 0.05^ij^	0.68 ± 0.07^f^	0.62 ± 0.01^i^	0.71 ± 0.06^de^
18	200 (+ 1)	300 (0)	20,000 (0)	80 (+ 1)	0.65 ± 0.04^gh^	0.71 ± 0.03^de^	0.78 ± 0.06^ab^	0.72 ± 0.01^d^	0.81 ± 0.02^a^
19	160 (−1)	200 (− 1)	20,000 (0)	70 (0)	0.46 ± 0.05^pq^	0.52 ± 0.04^n^	0.59 ± 0.08^jk^	0.54 ± 0.08^m^	0.63 ± 0.01^hi^
20	180 (0)	400 (+ 1)	16,000 (−1)	70 (0)	0.53 ± 0.02^mn^	0.59 ± 0.01^jk^	0.66 ± 0.05^g^	0.60 ± 0.04^j^	0.69 ± 0.08^ef^
21	180 (0)	300 (0)	24,000 (+ 1)	80 (+ 1)	0.62 ± 0.01^i^	0.68 ± 0.02^f^	0.75 ± 0.04^bc^	0.69 ± 0.08^ef^	0.78 ± 0.07^ab^
22	180 (0)	300 (0)	16,000 (−1)	60 (− 1)	0.45 ± 0.04^q^	0.51 ± 0.03^no^	0.58 ± 0.07^k^	0.52 ± 0.04^n^	0.61 ± 0.02^ij^
23	180 (0)	400 (+ 1)	24,000 (+ 1)	70 (0)	0.57 ± 0.06^kl^	0.63 ± 0.05^hi^	0.70 ± 0.06^e^	0.54 ± 0.03^m^	0.63 ± 0.02^hi^
24	160 (−1)	300 (0)	20,000 (0)	60 (−1)	0.42 ± 0.01^r^	0.48 ± 0.02^p^	0.55 ± 0.01^lm^	0.50 ± 0.08^o^	0.59 ± 0.07^jk^
25(C_5_)	180 (0)	300 (0)	20,000 (0)	70 (0)	0.53 ± 0.02^mn^	0.59 ± 0.04^jk^	0.66 ± 0.02^g^	0.60 ± 0.01^j^	0.69 ± 0.04^ef^
26	200 (+ 1)	300 (0)	16,000 (−1)	70 (0)	0.56 ± 0.05^l^	0.62 ± 0.04^i^	0.69 ± 0.03^ef^	0.63 ± 0.02^hi^	0.72 ± 0.01^d^
27	180 (0)	200 (−1)	24,000 (+ 1)	70 (0)	0.53 ± 0.02^mn^	0.59 ± 0.01^jk^	0.67 ± 0.02^fg^	0.60 ± 0.04^j^	0.69 ± 0.08^ef^
28	200 (+ 1)	200 (−1)	20,000 (0)	70 (0)	0.56 ± 0.05^l^	0.62 ± 0.04^i^	0.69 ± 0.08^ef^	0.63 ± 0.02^hi^	0.72 ± 0.01^d^
29	200 (+ 1)	300 (0)	20,000 (0)	60 (−1)	0.52 ± 0.01^n^	0.58 ± 0.02^k^	0.65 ± 0.04^gh^	0.69 ± 0.08^ef^	0.74 ± 0.03^c^

^C1,C2,C3,C4,C5^represent spraying drying process at center points

Experimental model = Box-Behnken design

Total number of spray drying treatments = 29

No of replicates = 03

^a-r^values with similar letters show homogenous group within row and column (p > 0.05)

The regression equations regarding the response factors at different days and storage intervals after spray drying process have been summarized in the Table 9. The optimized conditions of inlet air temperature (161–162 °C), feed flow rate (310–320 mL/hr), atomization speed (16550–16,600 rpm) and outlet air temperature (61–62 °C) were found for maximum retention of fatty acids at 25 °C after 60 days as ALA (123–124 mg/50 g egg), EPA (6.3–6.4 mg/50 g egg), DHA (11.6–11.8 mg/50 g egg), total omega-3 fatty acids (141–142 mg/50 g egg) and PV (0.60–0.61 meq/kg O_2_) of DEDP samples, respectively.

**Table 9:** Regression equations for response factors at different days and storage intervals after spray drying process

Response factor	Storage conditions	Regression equation
Powder Yield	at O day	Y = + 46.10 + 7.96X_1_ + 6.01X_2_ + 4.04X_3_ + 6.02X_4_ + 0.0250X_1_X_2_–0.0450X_1_X_3_ + 0.0253X_1_X_4_ + 0.0256X_2_X_3_ + 0.0251X_2_X_4_–6.10X_3_X_4_ + 0.9350X_1_^2^ + 2.92X_2_^2^ - 0.0525X_3_^2^ - 2.02X_4_^2^
ALA	at O day	Y = + 114.63–6.57X_1_ + 3.54X_2_–6.53X_3_–5.98X_4_–0.0925X_1_X_2_–0.2175X_1_X_3_ + 0.0975X_1_X_4_–0.04X_2_X_3_ + 0.2650X_2_X_4_–0.0650X_3_X_4_–0.5215X_1_^2^ + 0.4060X_2_^2^ + 0.4410X_3_^2^ + 1.03X_4_^2^
	after 3O days at 4 °C	Y = + 113.44–6.52X_1_ + 3.62X_2_–6.59X_3_–5.82X_4_–0.2325X_1_X_2_–0.1275X_1_X_3_ + 0.1775X_1_X_4_ + 0.0925X_2_X_3_ + 0.1250X_2_X_4_–0.6450X_3_X_4_–0.6762X_1_^2^ + 0.4050X_2_^2^ + 0.6250X_3_^2^ + 1.10X_4_^2^
	after 6O days at 4 °C	Y = + 110.35–6.59X_1_ + 3.57X_2_–6.62X_3_–5.88X_4_ + 0.0450X_1_X_2_–0.1150X_1_X_3_ + 0.1100X_1_X_4_ + 0.1250X_2_X_3−_ 0.0600X_2_X_4_–0.4825X_3_X_4_–0.6251X_1_^2^ + 0.5299X_2_^2^ + 0.6037X_3_^2^ + 1.33X_4_^2^
	after 3O days at 25 °C	Y = + 110.42–6.48X_1_ + 3.61X_2_–6.42X_3_–6.03X_4_–0.1200X_1_X_2_–0.0775X_1_X_3_ + 0.0750X_1_X_4_–0.0025X_2_X_3−_ 0.0425X_2_X_4_ + 0.0825X_3_X_4_–0.4374X_1_^2^ + 0.3963X_2_^2^ + 0.4776X_3_^2^ + 1.06X_4_^2^
	after 6O days at 25 °C	Y = + 105.27–6.46X_1_ + 3.46X_2_–6.48X_3_–5.96X_4_–0.1775X_1_X_2_ + 0.0002X_1_X_3_ + 0.1550X_1_X_4_ + 0.0400X_2_X_3−_ 0.0275X_2_X_4_ + 0.1400X_3_X_4_–0.2706X_1_^2^ + 0.5007X_2_^2^ + 0.6182X_3_^2^ + 1.21X_4_^2^
EPA	at O day	Y = + 13.10–0.7508X_1_ + 0.3867X_2_–0.7467X_3_–0.6608X_4_–0.0050X_1_X_2_–0.0150X_1_X_3_ + 0.0025X_1_X_4_–0.0100X_2_X_3_ + 0.0200X_2_X_4_ + 0.0150X_3_X_4_–0.0367X_1_^2^ + 0.0296X_2_^2^ - 0.0329X_3_^2^ + 0.0858X_4_^2^
	after 3O days at 4 °C	Y = + 12.12–0.7725X_1_ + 0.3467X_2_–0.7908X_3_–0.7067X_4_–0.0900X_1_X_2_–0.2600X_1_X_3_–0.2525X_1_X_4_–0.0800X_2_X_3−_ 0.1150X_2_X_4_–0.1925X_3_X_4_–0.1789X_1_^2^–0.1477X_2_^2^ - 0.1064X_3_^2^ – 0.0777X_4_^2^
	after 6O days at 4 °C	Y = + 8.84–0.8750X_1_ + 0.7558X_2_–0.4567X_3_–0.8092X_4_ + 0.2175X_1_X_2_–0.0425X_1_X_3_ + 0.0900X_1_X_4_–0.4625X_2_X_3−_ 0.0925X_2_X_4_–0.2350X_3_X_4_–0.2227X_1_^2^–0.8714X_2_^2^ - 0.4777X_3_^2^ – 0.0289X_4_^2^
	after 3O days at 25 °C	Y = + 9.80–0.8792X_1_ + 0.4425X_2_–0.8600X_3_–0.8983X_4_ + 0.1375X_1_X_2_ + 0.2050X_1_X_3_–0.4250X_1_X_4_ + 0.3325X_2_X_3−_ 0.0375X_2_X_4_–0.5825X_3_X_4_–0.2229X_1_^2^–0.3329X_2_^2^ - 0.4017X_3_^2^ – 0.1342X_4_^2^
	after 6O days at 25 °C	Y = + 4.60–0.8958X_1_ + 0.4550X_2_–0.8292X_3_–0.8583X_4_–0.1425X_1_X_2_ + 0.2800X_1_X_3_–0.2650X_1_X_4_ + 0.1775X_2_X_3−_ 0.3650X_2_X_4_–0.3250X_3_X_4_–0.2782X_1_^2^–0.2845X_2_^2^ - 0.1232X_3_^2^ + 0.0030X_4_^2^
DHA	at O day	Y = + 17.43–0.9983X_1_ + 0.4808X_2_–1.03X_3_–0.9300X_4_–0.0050X_1_X_2_ + 0.0200X_1_X_3_–0.0150X_1_X + 0.1000X_2_X_3−_ 0.0725X_2_X_4_–0.0175X_3_X_4_ + 0.0010X_1_^2^ + 0.1823X_2_^2^ + 0.0172X_3_^2^ + 0.1035X_4_^2^
	after 3O days at 4 °C	Y = + 16.16–1.07X_1_ + 0.4675X_2_–1.03X_3_–0.1.04X_4_ + 0.1175X_1_X_2_ + 0.0125X_1_X_3_ + 0.0450X_1_X - 0.0700X_2_X_3_ + 0.1650X_2_X_4_–0.0150X_3_X_4_ + 0.0808X_1_^2^ + 0.6771X_2_^2^ + 0.0596X_3_^2^ + 0.0308X_4_^2^
	after 6O days at 4 °C	Y = + 12.06–1.02X_1_ + 0.4983X_2_–0.9958X_3_–0.8708X_4_ + 0.0275X_1_X_2_ + 0.0350X_1_X_3_ + 0.0625X_1_X - 0.1425X_2_X_3_ + 0.1450X_2_X_4_–0.0800X_3_X_4_ + 0.0516X_1_^2^ + 0.9716X_2_^2^ - 0.0222X_3_^2^ + 0.0878X_4_^2^
	after 3O days at 25 °C	Y = + 13.48–1.07X_1_ + 0.5142X_2_–0.8950X_3_–0.9258X_4_–0.0025X_1_X_2_–0.0600X_1_X_3_ + 0.2975X_1_X - 0.2275X_2_X_3_ + 0.1125X_2_X_4_–0.1375X_3_X_4_ + 0.0131X_1_^2^ + 0.7218X_2_^2^ - 0.1794X_3_^2^ + 0.0593X_4_^2^
	after 6O days at 25 °C	Y = + 8.54–1.00X_1_ + 0.3583X_2_–0.9850X_3_–0.9058X_4_–0.0750X_1_X_2_–0.1450X_1_X_3_–0.0025X_1_X - 0.1750X_2_X_3−_ 0.0300X_2_X_4_–0.0950X_3_X_4_ + 0.1923X_1_^2^ + 0.5889X_2_^2^ + 0.0389X_3_^2^ + 0.0027X_4_^2^
TOFA	at O day	Y = + 145.32–8.32X_1_ + 5.24X_2_–8.30X_3_–6.77X_4_–0.0950X_1_X_2_ + 0.2125X_1_X_3_ + 0.0825X_1_X + 0.0600X_2_X_3_ + 2.70X_2_X_4_–0.0825X_3_X_4_–1.06X_1_^2^ + 1.36X_2_^2^ - 0.0837X_3_^2^ + 1.96X_4_^2^
	after 3O days at 4 °C	Y = + 141.69–8.36X_1_ + 4.43X_2_–8.42X_3_–7.56X_4_–0.2050X_1_X_2_ + 0.3750X_1_X_3_–0.0300X_1_X - 0.0575X_2_X_3_ + 0.1750X_2_X_4_–0.8275X_3_X_4_–0.7562X_1_^2^ + 0.9526X_2_^2^ + 0.5838X_3_^2^ + 1.06X_4_^2^
	after 6O days at 4 °C	Y = + 131.50–8.48X_1_ + 4.82X_2_–8.08X_3_–7.57X_4_ + 0.3000X_1_X_2_–0.1225X_1_X_3_ + 0.2675X_1_X - 0.4800X_2_X_3−_ 0.0075X_2_X_4_–0.7975X_3_X_4_–0.9235X_1_^2^ + 0.5053X_2_^2^ - 0.0260X_3_^2^ + 1.26X_4_^2^
	after 3O days at 25 °C	Y = + 134.70–8.43X_1_ + 4.53X_2_–8.25X_3_–7.96X_4_–0.0450X_1_X_2_ + 0.0675X_1_X_3_ + 0.0001X_1_X + 0.0650X_2_X_3_ + 0.0325X_2_X_4_–0.1400X_3_X_4_–1.19X_1_^2^ + 0.3461X_2_^2^ - 0.4489X_3_^2^ + 0.5311X_4_^2^
	after 6O days at 25 °C	Y = + 118.80–8.37X_1_ + 4.27X_2_–8.23X_3_–7.79X_4_–0.3950X_1_X_2_–0.4250X_1_X_3_–0.1125X_1_X + 0.0425X_2_X_3_ - 4225X_2_X_4_–0.1000X_3_X_4_–0.9022X_1_^2^ + 0.6441X_2_^2^ + 0.2828X_3_^2^ + 0.9691X_4_^2^
PV	at O day	Y = + 0.5300 + 0.0492X_1_ + 0.0192X_2_ + 0.0200X_3_ + 0.0650X_4_ + 0.0001X_1_X_2_–0.0025X_1_X_3_ + 0.0001X_1_X + 0.0025X_2_X_3_ + 0.0001X_2_X_4_ + 0.0001X_3_X_4_ + 0.0004X_1_^2^ + 0.0003X_2_^2^ + 0.0017X_3_^2^ + 0.0042X_4_^2^
	after 3O days at 4 °C	Y = + 0.5900 + 0.0500X_1_ + 0.0192X_2_ + 0.0183X_3_ + 0.0642X_4_–0.0025X_1_X_2_ + 0.0026X_1_X_3_ + 0.0002X_1_X + 0.0027X_2_X_3−_ 0.0028X_2_X_4_ + 0.0003X_3_X_4_–0.0025X_1_^2^ + 0.0013X_2_^2^ + 0.0001X_3_^2^ + 0.0062X_4_^2^
	after 6O days at 4 °C	Y = + 0.6600 + 0.0501X_1_ + 0.0175X_2_ + 0.0192X_3_ + 0.0650X_4_–0.0023X_1_X_2_ + 0.0025X_1_X_3_ + 0.0001X_1_X + 0.0002X_2_X_3_ + 0.0003X_2_X_4_ + 0.0001X_3_X_4_–0.0022X_1_^2^ + 0.0012X_2_^2^ + 0.0012X_3_^2^ + 0.0050X_4_^2^
	after 3O days at 25 °C	Y = + 0.6000 + 0.0558X_1_ + 0.0100X_2_ + 0.0117X_3_ + 0.0525X_4_ + 0.0025X_1_X_2_–0.0024X_1_X_3_–0.0225X_1_X - 0.0226X_2_X_3−_ 0.0100X_2_X_4_ + 0.0001X_3_X_4_ + 0.0146X_1_^2^–0.0117X_2_^2^ - 0.0116X_3_^2^ + 0.0171X_4_^2^
	after 6O days at 25 °C	Y = + 0.6900 + 0.0517X_1_ + 0.0083X_2_ + 0.0116X_3_ + 0.0600X_4_ + 0.0024X_1_X_2_–0.0050X_1_X_3_–0.0125X_1_X - 0.0200X_2_X_3−_ 0.0025X_2_X_4_ + 0.0002X_3_X_4_ + 0.0113X_1_^2^–0.0087X_2_^2^ - 0.0088X_3_^2^ + 0.0137X_4_^2^

X_1_ = Inlet air temperature; X_2_ = Feed flow rate; X_3_ = Atomization speed; X_4_ = Outlet air temperature

*ALA* Alpha-linolenic fatty acids, *EPA* Eicosapentaenoic fatty acids, *DHA* Docosahexaenoic fatty acids, *TOFA* Total omega-3 fatty acids, *PV* Peroxide value

Several authors, in accordance with our results, report the effect of storage temperature on egg powder fatty acids composition. Deslypere et al. [24] results also conclude that storage at lower temperatures for several months yielded no perceptible changes in n-3 PUFAs of fat tissue aspirates which is compatible with our results. This study showed that PUFAs loses increases with storage which is in accordance with observation of Terao et al. [25] that egg lipids underwent high oxidation during spray-drying; moreover, they observed that this oxidation significantly increases during storage (1 and 3 months). Furthermore, several previous research studies described monounsaturated fatty acid and PUFAs losses during extensive heat processing [26–28]. In addition, some studies have been focused only on n-3 PUFAs losses because of their high nutritional relevance [29].

In the case of DEDP, the loss of essential fatty acids was already predicted because the PUFA_S_ content were high in DEDP samples and the heat treatment applied was severe. The thermal effects could be clearly observed at high outlet air temperatures, in accordance with other published reports. With increase of temperature, retention of PUFAs decreases and browning of powder increased, but lower temperature cause retention of moisture and low-quality powder with increased drying time [30, 31]. High temperature treatment causes protein denaturation and modifies lipoprotein structure. This change leads to decreased oxidative stability of egg lipids [32]. Higher temperature conditions in spray dryer causes higher losses of omega-6 and omega-3 PUFAs and also less favorable to omega-6/omega-3 and PUFA/SFA ratios. Mostly C_20:4n-6_ and C_22:6n-3_ PUFAs are destroyed at high temperature [33].

The safety and quality of powdered eggs depend on at least two critical steps as the drying process itself and the storage conditions such as length and temperature. The drying process uses high temperatures that can accelerate reactions between lipids and molecular oxygen, resulting in losses of nutritional and sensory properties of egg products. At the same time, there is an increasing interest on the consumption of food that have a higher content of omega-3 PUFAs than conventional foods. However, this increase in the unsaturation of fatty acid can lead to an increase of lipid oxidation, especially during the drying process or the storage.

Unsaturated fatty acid losses have been widely reported as an indicator of lipid oxidation. As a rule, in foods, susceptibility to oxidation of phospholipids increases with the unsaturation [34]. The spray-dried eggs are highly oxidized and very susceptible to oxidation in comparison with raw eggs [35]. This fact is related to the structure of phospholipids in the raw yolk that protect against oxidation. Phospholipids are interwoven in the exterior structure of low-density lipoprotein and this compact surface prevents the contact of oxygen with the lipid core of the particle [36].

Egg powder was produced under high temperature scales, which led to many changes in egg components, resulting in lower retention of PUFAs in DEDP samples during storage. Food industries of using spray dried omega food materials are facing the problem of oxidation as these possessed unstable PUFAs during processing and storage. Several appropriate methods have been applied to reduce or prevent lipid oxidation of spray dried powders in order to improve final functional food quality. The most commonly used method is the addition of antioxidants. A combination of antioxidants with inert gas packaging can strongly stabilize the spray dried omega food products. Major finding supports that spray drying of whole egg at moderate conditions of air inlet temperature, feed flow rate, atomization speed and outlet air temperature resulted in a product that enhanced considerably the retention of PUFAs and good quality powder that could further be used for development of functional food products. Thereby, it could be concluded that slight lipid oxidation mostly occurs during spray-drying but this oxidation rate may be enhanced during storage. So, care should be taken during storage of DEDP samples.

## Conclusion

The results of present study demonstrated the optimized conditions of inlet air temperature (198–199 °C), feed flow rate (398–399 mL/hr), atomization speed (16000–16,010 rpm) and outlet air temperature (76–77 °C) for maximum yield of designer egg dried powder samples (66.20 ± 0.20 g/500 mL). The inlet and outlet air temperature were seen to be as major factors affecting the essential fatty acids content in spray dried samples. Furthermore, the results from this work will aid in the formulation of healthy food products supplemented with designer egg dried powder and may address a critical industrial demand in terms of formulation options. Additional studies should be undertaken to enhance the shelf life of omega food products by supplementation of antioxidants and gradual reduction of oxidation process. Furthermore, future studies should focus on treatment of nutritional disorders through the functional foods and their absorption, metabolism and distribution pattern into biological tissues.
